# Spanning maternal, newborn and child health (MNCH) and health systems research boundaries: conducive and limiting health systems factors to improving MNCH outcomes in West Africa

**DOI:** 10.1186/s12961-017-0212-x

**Published:** 2017-07-12

**Authors:** Irene Akua Agyepong, Aku Kwamie, Edith Frimpong, Selina Defor, Abdallah Ibrahim, Genevieve C. Aryeetey, Virgil Lokossou, Issiaka Sombie

**Affiliations:** 1 Ghana Health Service, Research and Development Division, P.O. Box MB-190, Greater Accra region, Ghana; 2 University of Ghana School of Public Health, P.O. Box LG13, Legon, Accra, Ghana; 3 West African Health Organization, Bobo-Dioulasso, 01BP 153 Bobo-Dioulasso 01, Burkina Faso

**Keywords:** Maternal newborn and child health, Health systems, Context, Boundaries, Complexity, ECOWAS

## Abstract

**Background:**

Despite improvements over time, West Africa lags behind global as well as sub-Saharan averages in its maternal, newborn and child health (MNCH) outcomes. This is despite the availability of an increasing body of knowledge on interventions that improve such outcomes. Beyond our knowledge of what interventions work, insights are needed on others factors that facilitate or inhibit MNCH outcome improvement. This study aimed to explore health system factors conducive or limiting to MNCH policy and programme implementation and outcomes in West Africa, and how and why they work in context.

**Methods:**

We conducted a mixed methods multi-country case study focusing predominantly, but not exclusively, on the six West African countries (Burkina Faso, Benin, Mali, Senegal, Nigeria and Ghana) of the Innovating for Maternal and Child Health in Africa initiative. Data collection involved non-exhaustive review of grey and published literature, and 48 key informant interviews. We validated our findings and conclusions at two separate multi-stakeholder meetings organised by the West African Health Organization. To guide our data collection and analysis, we developed a unique theoretical framework of the link between health systems and MNCH, in which we conceptualised health systems as the foundations, pillars and roofing of a shelter for MNCH, and context as the ground on which the foundation is laid.

**Results:**

A multitude of MNCH policies and interventions were being piloted, researched or implemented at scale in the sub-region, most of which faced multiple interacting conducive and limiting health system factors to effective implementation, as well as contextual challenges. Context acted through its effect on health system factors as well as on the social determinants of health.

**Conclusions:**

To accelerate and sustain improvements in MNCH outcomes in West Africa, an integrated approach to research and practice of simultaneously addressing health systems and contextual factors alongside MNCH service delivery interventions is needed. This requires multi-level, multi-sectoral and multi-stakeholder engagement approaches that span current geographical, language, research and practice community boundaries in West Africa, and effectively link the efforts of actors interested in health systems strengthening with those of actors interested in MNCH outcome improvement.

## Background

The countries of the Economic Community of West African States (ECOWAS) make up most of the West African sub-region, and together have an estimated population of almost 350 million. ECOWAS comprises 15 countries, all of which are classified as low or lower middle income. The sub-region is home to an immense diversity of peoples, cultures, languages and religion. Layered on traditional ethnic, religious and language diversity and further increasing complexity is the colonial legacy of fragmentation by official language into Anglophone, Francophone and Lusophone.

Political, social and economic stability in the sub-region has been marred in the past by coups, internal civil strife and structural adjustment. More recently, instability has included terrorist threats such as Boko Haram and Al-Qaeda in the Maghreb, climate-linked food insecurity, increased use of some countries as a transit route for drug trafficking, and the most massive outbreak of Ebola virus on record, which spanned the borders of three countries and led to imported cases to three more countries in the sub-region and beyond. Despite its myriad of challenges, the ECOWAS is also a sub-region of achievement and potential. Its population is young and many of its economies are growing. Multi-party democracy and political stability are slowly becoming the story of the present.

Current maternal and under five mortality rates in the ECOWAS are the results of a slow progressive decline from the levels of previous decades, and are clearly moving in the right direction. However, in relative terms, these rates lag behind global as well as sub-Saharan averages [1]. Neonatal mortality ratios have been estimated as high as 182/1000 in Sierra Leone, 128/1000 in Mali and 124/1000 in Nigeria. Maternal mortality ratio estimates are similarly high in Cote D’Ivoire (720/100,000), in Niger (630/100,000) and in The Gambia (430/100,000) [2]. Additionally, within these global average mortality trends for West Africa are wide national and sub-national variations [3–7]. This raises the critical question of why maternal, newborn and child health (MNCH) mortality improvements in West Africa are lagging behind sub-Saharan African and global averages.

There is a large and increasing body of research on effective interventions for MNCH improvement [8]. If knowledge about proven effective interventions alone were enough, West Africa should not have its current and persisting challenges. Clearly, there are other factors affecting the ability to implement these interventions to scale, sustain implementation and accelerate improvements in MNCH outcomes.

One such cluster of factors that merits closer study is the link between health systems, MNCH interventions and outcomes. Weak health systems are one of several factors blamed for slow progress towards attainment of health-related goals in sub-Saharan Africa [9, 10]. Interventions to improve MNCH outcomes are implemented within health systems. What factors within these systems are conducive or limiting to the effective implementation at scale of these interventions? As health systems exist within a global, national and sub-national context, does this context also influence effective implementation to scale of MNCH interventions?

Sheikh et al. [11] note the somewhat fragmented nature of global health with multiple territories of practice and frames and call for the practice of global health to include efforts to bridge these boundaries if it is to become more relevant for the communities that are its intended beneficiaries. The fields of MNCH research and of health policy and systems research are two of the territories in global health that would benefit from such “*reaching across borders … to build relationships, interconnections and interdependence*” [12], or boundary spanning efforts.

The current study therefore aimed to explore health system factors conducive or limiting to MNCH policy and programme implementation to scale for health outcome improvement in the ECOWAS. It also looked at how and why these factors work or may work in context to influence MNCH outcomes.

## Methods

The study design was a mixed methods case study of MNCH and health systems in West Africa. Data collection involved a non-exhaustive desk review of grey and published literature and key informant (KI) interviews. Within West Africa, we focused particularly, but not exclusively, on the six target countries of the Innovating for Maternal and Child Health in Africa initiative, namely Benin, Burkina Faso, Ghana, Mali, Nigeria and Senegal. These countries together hold approximately three quarters of West Africa’s population. They include Anglophone (Nigeria and Ghana) as well as Francophone (Benin, Burkina Faso, Mali and Senegal) countries and represent the geographical spread from the Southern coastal belt through to the Northern savannah of West Africa.

The desk review focused on scoping already existing research, and we searched the published and grey Anglophone and Francophone health systems research and MNCH research as well as policy, programme and implementation documents from West Africa, in general, and the six target countries in particular, over the period 1990–2015. Databases searched online were PubMed, Scopus, ScoIndex, CAIRN.Info, CINHAL, Google Scholar, Africa Journals online, JSTOR, Embase, the Cochrane Library and EPPI-Centre. We also asked KIs for links to relevant grey literature such as annual reports, programmes of work, aide-mémoires and performance reviews. Documents obtained and reviewed included peer-reviewed publications, systematic reviews, country reports, and research project, agency and non-governmental organisation (NGO) documents and reports.

Online search terms included maternal health; antenatal care; postnatal care; newborn health OR neonatal health; child health OR under-five health; family planning; reproductive health; abortion; maternal mortality; maternal morbidity; neonatal mortality; infant mortality AND human resources OR nurs* OR midwif* OR health workforce; governance, leadership, management; accountability; drugs, medicines, technologies; commodities; health information systems; service delivery; financing OR fee exemption OR health insurance AND transport; ambulance, community; birth preparedness AND Benin, Burkina Faso, Ghana, Mali, Nigeria, Senegal, West Africa AND power, trust, decision making, policy, stakeholders, politics, context.

The KI interviews aimed to complement as well as fill gaps in the literature review. Selection of respondents was purposive to ensure representation of national and sub-national key actors and stakeholders as well as development partners involved in health systems and/or MNCH in the six target countries. We relied on a contact list provided by the West African Health Organization (WAHO) of potential actors and stakeholders meeting these inclusion criteria in the six focus countries. WAHO engages with the health sectors and ministries of health of all the 15 countries of the ECOWAS and was therefore uniquely placed to help identify key respondents within countries. The final list of interviewees was conveniently based on who could be reached with our initial exploratory phone calls and email, and was available and willing to be interviewed face-to-face, by email or skype. We stopped interviewing when no new issues and perspectives arose in the interviews. We conducted a total of 48 KI interviews.

All interviews were conducted between December 21, 2015, and February 2, 2016, by the same two team members (one Anglophone and one bilingual). The KI occupied positions such as country director, director general, national advisor, divisional or sub-divisional director, senior advisor, specialist and consultant. The background of KIs is summarised in Table 1.

**Table 1:** Summary background of key informants (*n* = 48)

Sex	*n* %	
Female	23	48%
Male	25	52%
Agency of work		
Development partner (e.g. USAID, UNFPA, WHO, UNICEF)	15	31%
Ministry of Health/Public sector agency, e.g. Ghana Health Service	17	35%
NGO (National, e.g. Federation of Muslim Women, Members of Coalition of NGO in Health, and international, e.g. Family Care, Health Keepers, Marie Stoppes)	9	19%
Professional association representative	3	6%
Clinical Specialist, e.g. Obstetrician, Gynaecologist, Paediatrician	3	6%
University Researcher (one respondent held a dual position in the Ministry of Health and in a University)	2	4%
Country		
Ghana	17	35%
Nigeria	5	10%
Burkina	8	17%
Benin	6	13%
Mali	6	13%
Senegal	6	13%

All interviews were performed with informed consent. Where permission was given, interviews were recorded and transcribed. Where KIs declined to be recorded, we relied on the notes taken by the interviewer.

A WAHO country meeting in Ghana in November 2015 and a sub-regional meeting in Dakar in February 2016 were used as opportunities to present findings and preliminary conclusions for validation, critique and input of multi-level health sector stakeholders from countries in the sub-region participating in the meetings.

### Conceptual framework for the study, topic areas for exploration and data analysis

Topic areas for exploration were derived from a unique conceptual framework developed of how health systems relate to MNCH interventions and outcomes. We developed the framework through a review of several existing frameworks proposed for describing and analysing health systems [13–20] as well as through brainstorming. Across these frameworks, the ‘health’ of individuals through life often measured in terms of fatal and non-fatal outcomes is an intrinsic goal, and the defining goal of health systems.

Beyond this defining goal, two other goals commonly appear as intrinsic in most, though not all, frameworks in the literature, namely responsiveness and fairness in financial contribution or financial risk protection. Responsiveness relates to the legitimate expectations of individuals regarding the non-health aspects of the health systems such as how people are treated (dignity, respect, autonomy, choice, confidentiality) and the environment in which they are treated when seeking healthcare [21, 22]. Responsiveness is a social goal, and one that is not necessarily unique to health systems. The health system, like other social systems such as justice and education, is expected, beyond its core goal, to also meet this common social goal. Fairness in financial contribution or financial risk protection has gained increasing prominence with the push towards universal health coverage. It refers to ensuring that paying for healthcare does not itself expose people to undue risk by being catastrophic.

Key in determining the ability of health systems to deliver intrinsic goals are what are commonly described by health systems frameworks as building blocks or hardware. These include resources (human, infrastructure, equipment, tools and supplies), financing arrangements, heath information systems, governance or stewardship, medicines and technology.

Also increasingly recognised as key determinants of the functioning of health systems, are the people within them [23], their power and how they chose to exercise it, and the processes they set up and operate to run the health system. This aspect of health systems is sometimes described as the software. People are central to and drive health systems, similar to the way in which software drives computer systems. Depending on the operating systems and software, the same computer hardware can perform differing functions leading to differing outputs. In health systems, depending on people, their power, interests, networks, relationships, etc., there will be variation in performance even with the same hardware or building blocks.

**Fig. 1:**
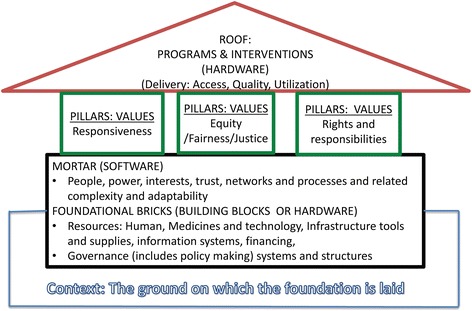
Conceptual framework – The health system as a shelter

Drawing on, synthesising and critically reflecting on these existing frameworks and how they relate to MNCH interventions and outcomes, we conceptualised health systems as a house that shelters the health of the population, including mothers, newborns and children (Fig. 1).

### Foundational bricks: classical health system building blocks (hardware)

Within this framework, what are popularly referred to as the health system building blocks are conceptualised as the foundational bricks of the house. The strength and ability of these bricks to support the structure and withstand routine shocks as well as in severe crisis (resilience), contributes to the strength of the health system.

### Mortar: people, process, power (software)

What is referred to as the software in some health systems models (people, processes, power, etc.) we consider as the mortar that determines how well the foundational bricks hold together. They therefore also contribute to the strength, stability and resilience of the health system. Power and influence in organisations are “*the capacity to effect (or affect) organizational outcomes*” and “*to get desired things done, to effect outcomes – actions and the decisions that precede them*” [24]. People as actors and stakeholders are the drivers of decisions and implementation – who has power or influence to do what, as well as how they use their power or the manifestations of their power, matters. Who has power over who and what, who has power with whom and/or through whom are all critical and important limiting and conducive factors to the implementation and outcomes of MNCH interventions and programmes. Networks, intelligence and information, transparency, trust and respect, conflict, stakeholder interests, and perspectives are all part of the human interactions and processes that form the mortar.

### Pillars: values

Every health system has explicit and implicit underlying values that influence decisions and actions. For example, the design of a health system, such as financing arrangements and human resource distribution in a system where health is explicitly and implicitly recognised as a fundamental human right and equity has a high premium, can be very different from that in a health system where it is not. We considered the underlying values of health systems to be the pillars of the house or shelter.

We considered three key pillars or underlying values in health systems as responsiveness, equity (fairness and justice), and rights and responsibilities. Although typically, in health systems frameworks, responsiveness is described as an intrinsic outcome, here, responsiveness is a value as well as an intrinsic outcome. We chose to place it predominantly as a value in this framework since we think values drive and shape the outcomes of health systems. The treatment of people – whether clients (external customers) or health workers (internal customers) – with dignity, respect, confidentiality, autonomy, and prompt attention, as well as social support networks and choice [21], are an outcome of health systems. However, they are also a manifestation of the values placed on human rights in any given system. Thus, they reflect value as well as outcome. Equity or justice and fairness are concepts with similar meanings and we use them interchangeably. They relate to being impartial, acting with integrity and rightness, and the awarding of what is due in the distribution of resources, gains, losses, rewards and punishment in societies and social relationships. Equity, like responsiveness, is a value and a process as well as an outcome [25]. Who has the right to what, how and why, and the related concept of who is responsible for what, how and why are values within health systems that will influence how people are treated as well as accountability.

### Roof: interventions and programmes

We conceptualised interventions and programmes as the roof of the shelter. The foundation and pillars support this roof to serve the population. They influence the feasibility, effectiveness and outcome of implementation of these interventions. This includes interventions to improve the health of mothers, newborns, infants, children and adolescents. Supporting interventions and neglecting the health system and its values is putting up a structure with weak foundations and pillars to support the roof. It will lack resilience and will be liable to collapse when exposed to stress.

### The ground on which health systems are built: context

By context we refer to “*the set of circumstances or facts that surround an event, situation, etc*.” [26]. As the setting within which health systems exist and events occur, context needs to be considered to fully understand the situation, phenomenon or events of interest. Staying with our framework image of the health system as a shelter, context can be seen as the ground on which the foundation is laid.

Types of context, or the surrounding circumstances within which health systems develop and function, are multi-layered, from global to national through to sub-national. They include demographic patterns and trends, sociocultural factors, including societal values and norms, macroeconomic conditions and trends, history, politics and ideology [27, 28]. Context is a critical influence on the stability and resilience of health systems. Like the biblical parable of the house on sand and the house on rock, health systems in weak contexts can be houses built on sand, liable to cave in when exposed to stress. A recent example in West Africa is the devastating effect of the outbreak of Ebola in Guinea, Liberia and Sierra Leone, all of which were fragile and/or post-conflict states with histories of economic and human underdevelopment [29]. The health systems weaknesses that Ebola exposed were in part due to these severe contextual challenges.

We analysed our data by the themes or categories in this conceptual framework. All data analysis was performed manually, and we looked for commonalities, as well as any contrasts and contradictions. We triangulated the desk review and the KI data as part of quality and internal validity checking.

### Limitations of the framework

Our framework is a useful device to help organise enquiry and analysis, but like most frameworks, it has its limits. In practice, it is almost impossible to make a sharp delineation between processes and the structures, institutions and actors who initiate, maintain and terminate them. Processes within health systems are driven by people and use, and are influenced by the building blocks or hardware and the context. Processes occur within and between structures and institutions.

## Results and discussion

The results section simultaneously presents and, as relevant, compares and contrasts the results from the desk review and the KI interviews. We first present our findings in a more descriptive form using the categories in the framework. However, the inter-relatedness between the categories means that, to avoid repetition, it is not possible to draw neat lines. This inter-relatedness presents the more complete explanation of how and why the health systems and contextual factors worked to affected MNCH policy and programme implementation and outcomes. At the end, we therefore describe a simple framework for this inter-connectedness developed from our findings and illustrate it using examples from the data.

### Programmes and interventions (service delivery): the roof

Through the desk review and the KI interviews we identified several interventions being piloted, researched or implemented to scale in several countries to improve MNCH outcomes in the ECOWAS. The variety of depth, breath and manner of implementation were such that to map them in detail would require a separate study. They can be summarised as interventions related to various kinds of mortality audits (community/verbal, facility-based reviews and confidential enquiries into maternal deaths); near miss audits; criterion-based clinical audits; referral; electronic/mobile health; and financing, task shifting and preventive care such as antenatal, postnatal and family planning. Financing interventions included out-of-pocket user fees introduction, reduction or removal; exemptions such as free caesarean section, antenatal care delivery, or a combination; and community-based and national health insurance.

Access to and utilisation of these interventions was a conducive (adequate access, utilisation) as well as a limiting (inadequate access, non-utilisation) factor from the desk review findings. Access to MNCH interventions was influenced by geographic factors such as the location and distance of facilities from households and communities, as seen in several studies from Ghana and Burkina Faso [30–34]. Sometimes, even where service was geographically accessible, the content of the service and the lack of particular types and levels of service proved to be limiting [35, 36].

Content of service also had an influence on the quality of care through its effect on clusters of factors related to other health system factors such as human resource quantity and quality, infrastructure, tools and supplies, as well as procedural and capacity challenges. These influenced care during referral transportation, delays in care on arrival and service delivery procedures. They also affected the expression of values such as responsiveness.

“*The logistics to work with, and then also infrastructure, electricity, water, privacy they are all not adequate.*” (Public sector health director, Ghana)

“*The setup of the health facilities makes it even more difficult to guarantee confidentiality. Consulting spaces are not secured so you have people walking in and out during patient consultations, right from interrogation through diagnosis to prescribing treatment. This is why most patients prefer to go to private facilities where the minimum anonymity and confidentiality could be guaranteed.*” (National level director, Ministry of Health, Mali)

Addressing these problems was conducive to MNCH service delivery. For example, it was observed in rural Burkina Faso that provision of infrastructure, equipment, tools and supplies improved MNCH service access and utilisation [37]. In Nigeria, ensuring adequate allocation of resources to execute identified gaps in the health and social sectors was conducive to the establishment of maternal death reviews [38].

Conducive and limiting health system factors to access and utilisation of interventions were sometimes closely linked to other sectors and contextual factors. For example, apart from ambulance availability [39], referral transportation was also facilitated or inhibited by the quality of roads and communication systems, such as mobile phones, that enabled communication between health workers performing different tasks in the same or different facilities [39]. A systematic review of all published mortality audits in low- and lower middle-income countries (1965–2011) to identify the most frequent avoidable factors in childbirth-related deaths [40], found that, in 7% of cases, factors related to transportation, such as lack or delay in transport, poor transport between facilities and from home to facility, contributed to the death.

Limiting factors mentioned by KIs were similar to those emerging from the desk review. They included quality of care, policies that influence service delivery not being adequately translated into real work, geographic location, distribution of services including inequities in distribution, and lack of functional primary healthcare arrangements, including referral systems and pathways.

“*… people are deprived of services, so there needs to be some kind of primary health care system so that the different levels of health care are interconnected, … there should be a referral pathway to different levels of health system, … or cheap transportation when there is the need … for referral of people who have complications either during pregnancy or child birth …*” (Midwifery consultant, Ghana)

“*… people they don’t have access to either free or cheap transportation when there is a need for referral of people who have complications either pregnancy or childbirth …*” (Multilateral development partner, Ghana)

Keeping interventions up to date with current knowledge was sometimes a challenge to programmes and interventions.

“*Emerging MNCH related services don’t get introduced until later because national protocols and policy frameworks are not regularly updated to reflect current research finding and WHO standards.*” (Bilateral development partner, Senegal)

Similarly, getting interventions into actual implementation versus just having them on the policy agenda or even moved beyond the agenda into formulation was sometimes a challenge.

“*We need to do a lot more in the area of translating policies into actual implementation at the service delivery points …*” (National health director, Nigeria)

“*I’m personally involved in a maternal mortality monitoring system which is supposed generate data regarding the death of women. This data is supposed to be analysed and reintroduced into national information system to enhance the health system and inform the programming of interventions to improve service targeted at mothers and children, but this activity has not seen the light of day despite all the trainings that have been held in practically all the districts, not to talk of all the laws voted to this effect.*” (Head of an academic department, Benin)

Conducive factors identified were generally the opposite of the limiting factors, such as availability of infrastructure or an ambulance service.

“*… we have more than 35,000 health facilities; primary secondary and tertiary health facilities, and they are all spread across the country. … the fact that they are available, and that a large percentage of them do render services is a conducive factor, we need to strengthen this*” (Programme Director, Nigeria)

### Health systems values: the pillars

“*One of the weaknesses of our health system is the lack of respect for the rights of our facility users, the conditions of hospitalization and consultation leaves much to be desired; the settings do not guarantee the minimum privacy the patients require. Added to the unsuitable setting is also the issue of poor reception from the service providers.*” (Representative of a professional association, Burkina Faso)

Poor responsiveness to clients appeared as a limiting factor in the desk review as well as the KI interviews. Several papers as well as KI interview respondents reported on client experiences of intimidation and being scolded, limited choices, ‘silent’ treatment and lack of privacy. Negative provider attitudes towards clients and their effects on service use were also reported. For example, poor skilled birth attendant attitudes were reported as a reason why mothers preferred home births and refused skilled attendants. There were also positive reports of responsive services, with good attitude of healthcare providers towards the client and availability of caring midwives at health facilities listed as reasons for facility-based care seeking [32]. These acted as conducive factors.

Apart from poor responsiveness to clients, poor responsiveness of organisations to the workers (internal clients or customers) also appeared to be an issue. A study in Ghana found that perceptions of unfairness in organisational relationships and processes and a sense that the organisation did not treat health workers with the respect and care they expected was a source of demotivation that spilled over to affect quality of care [41]. Conflicts between staff also worked to demotivate them and negatively affect the quality of care [42].

Research into equity, justice or fairness as a value in health systems in West Africa and the impacts of the extent to which it is held as a value on MNCH interventions, service delivery and outcomes was a major gap area in the literature. The papers we reviewed had generally researched equity and reported on it as an outcome, rather than as a value. For example, several papers reported that inequities in access and in financing, such as fee removal, disproportionally benefited the wealthier groups in Mali. Women in the poorest income group were less likely to be insured, despite the modest and heavily subsidised enrolment, and the richest households had a greater decline in out-of-pocket payments with the introduction of health insurance. However, it could be deduced that equity, as a value, was implicit in the myriad of fee exemptions, community-based health insurance and national health insurance scheme (NHIS) policies that had been and were still being tried in several countries in the sub-region to improve access for the poor.

Similarly, in the KI interviews, the idea of equity, justice or fairness as values that may explicitly underpin health systems and have an impact on decisions and outcomes did not appear to be something that had received the level of attention that other issues, such as interventions, service delivery and health system building blocks, had. Implicitly, however, it appeared to be there, including when a respondent mentioned that there was sometimes a clash of values between more economically oriented efficiency perspectives and more socially oriented equity and effectiveness perspectives.

“*The by-in from health system’s financial controllers is very critical. However, they do not always appreciate the fact that the health system is a social organization that provides health care to its population based on social tariff which may not easily lend itself to cost recovery.*” (Regional level director, Burkina Faso)

There was similarly a scarcity of literature that dealt with rights, responsibilities and the related issues of accountability. Specifically, we refer to who is responsible for what and to whom do they give account. We also refer to who has the right to what, why and how are these rights considered within health systems in West Africa and the effects on MNCH outcomes. However, some of the issues emerged indirectly in KI interviews.

“*Organizational wise, everything is in place, we have a good organizational structure at all the levels and we are backed by appropriate framework and policy but they are ineffective and they lack accountability … no one is accountable to anyone*” (Local NGO staff, Ghana)

## Foundational bricks (building blocks or hardware)

### Governance

Governance deals with how decision-making is organised and shared across health system-levels (national, sub-national, hospital, health centre and community), management and leadership capacities for MNCH policies and programme development, and implementation and existing accountability mechanisms in country for health systems and MNCH. The desk review as well as the KI interviews highlighted a wide range of limiting factors related to governance. These included priority setting, vision and leadership capacity. Conversely, improved leadership and other capacities were found to be conducive [43].

“*Misplaced priority by the governance: sometimes there are more serious issues to be done but then actually the governance is doing something else, when the communities are looking for something else and so on, so there is a misplaced priority.*” (Local NGO staff, Nigeria)

“*… some don’t have any vision, it’s just because they have been appointed to be there …* [they have] *inadequate knowledge and skills to be in a leadership position. People have not been trained to be in a leadership position and they are there, so they are unable to perform.*” (National health director, Ghana)

Institutional power hierarchies were reported to be strong in several countries and affected decision-making and implementation in various ways. For example, one of the conclusions of work in Senegal to identify barriers to, and facilitators of implementation of facility-based maternal death reviews was that institutional leadership and hierarchy affected the implementation and outcomes of the intervention. Specifically, non-participation of the head of department in the audit meetings and lack of feedback to staff who did not attend the audit meetings were identified as barriers. Strong traditional hierarchies in the relationship between doctors and other categories of personnel acted as a barrier to the establishment of multi-disciplinary teams. Conversely, the main facilitators were involvement of the head of the maternity unit, acting as a moderator during the audit meetings, and participation of managers in the audit session to plan appropriate and realistic actions to prevent other maternal deaths [44].

The nature and depth of implementation of decentralisation and insufficient decentralised decision-making authority acted as a limiting factor through its effect on the ability of mid and floor level managers to respond flexibly and appropriately in context to effectively implementing policies and programmes. Work in Ghana on district manager decision space showed that hierarchical authority and resource uncertainty constrained district manager decision space. These constraints gave rise to a leadership type oriented toward serving the bureaucratic functions of the health system. As a result, district-level management and leadership were sometimes constrained in their ability to respond to MNCH service delivery challenges [45].

In a study of the impact of decentralisation on sexual and reproductive health services in Ghana, Mayhew [46] found that, while some decision-making about resource allocation was meant to take place at district and regional level, in practice, it remained centrally controlled. Though this might have been a necessary safeguard for sexual and reproductive health services, it also hindered aspects of implementation at the local level.

However, decentralisation did not always have a positive effect on outcomes. Abimbola et al. [47] found that decentralisation in Nigeria negatively influenced the retention of rural health workers in two ways. Firstly, the salary of primary healthcare (PHC) workers was often delayed and irregular because of delays in transfer of funds from the national to sub-national governments. Secondly, the primary responsibility for PHC was often left to the weakest tier of government, namely local governments. The result was that rural PHC workers were attracted to working at secondary levels of care run by the state government and tertiary levels run by the federal government. These were often in urban areas where salaries were higher and more regular.

There could also be complex challenges with uniformity in national policy and programme acceptance and implementation depending on the model of decentralisation.

“*… Another bottleneck is the complex governance system … in Nigeria it is not unitary, so it’s not mandatory for a State to keep into the national health policies, we have to do a lot of advocacy to ensure buy-in.*” (National health director, Nigeria)

Floor or facility level governance issues such as leadership, interpersonal and interprofessional relations, conflicts among staff, higher level officials’ failure to adequately recognise, acknowledge and deal with the frontline worker resource availability, motivation and conflict also affected implementation of interventions and programmes [42].

“*… the important ingredient for strengthening health system development will be harmony across the cadres. There is largely a lot of disharmony in that sector and it impacts negatively on health systems development so we need to have a way of ensuring industrial harmony, we need to also ensure accountability in service delivery.*” (Head of department, Nigeria)

Public accountability of those who decide and act also emerged as a conducive or limiting factor depending on the circumstances. Lodenstien and Dao [48] found in rural Mali that, if decentralisation policies do not address public accountability, they will not fundamentally change human resource management, quality and equity of staffing. KIs also commented on accountability and the related issue of corruption.

“*Regarding accountability, I’ll sincerely admit that the absence of the requisite tools and relevant accountability mechanisms within the health system makes it difficult for people to be answerable even if they are willing to do so.*” (Representative of an NGO, Burkina Faso)

“*… then the issue of corruption which whether we like it or not is a canker among our people.*” (Local NGO, Nigeria)

“*Corruption and nepotism is bedevilling the health system in Mali. You know, a Director needs people and competent people for that matter to get things done but you just can’t appoint or recruit people, no matter their competence. As a director, I know what I want in a head of department but all efforts to have certain people to occupy certain positions have not yielded much fruit for political reasons. Most people occupying positions within the health system are there for political reasons, not because of their competence*.” (National level director, Ministry of Health, Mali)

Regulation and accreditation of healthcare providers, including providers of MNCH, also appeared to be an area of some weakness in governance.

“*Because we do not have any accreditation system, service providers do not really see the need to update their knowledge, some have never undergone any additional training since they graduated.*” (Head of an academic department, Burkina Faso)

Conducive governance factors included softened institutional power hierarchies, egalitarian team functioning such as shared decision-making and responsibility for results, facilitation of local innovation and continuous improvement, and multi-stakeholder, multi-level participation in governance to improve decision-making and strong and functional accountability arrangements.

### Medicines and technologies

Conducive and limiting factors influencing the availability and use of medicines and technologies included supply chain, quality of medicines and the related issues of storage conditions for essential medicines.

There were some success stories in the sub-region.

“*The UN life-saving commodities programme is a very good conducive factor, if countries in the region can now adopt it, Nigeria has adopted it making sure that these commodities are available … Government, working with partners, have made a commitment to make family planning commodities which are some of the life-saving commodities free and are available to all. … there is local production of Chlorhexidine so that’s also one of the life-saving commodities in Nigeria.*” (Head of department, Nigeria)

Unfortunately, as with many of the health system factors, the more common stories this study unearthed were challenges related to shortages, inadequacies, non-availability of essential medicines, tools and supplies (including blood), lack of technologies, and problems with infrastructure.

Information, communication and technology-related interventions are of increasing importance in the West African sub-region in small and large scale pilot projects. A systematic review of the role of mobile health interventions targeting healthcare workers in improving pregnancy outcomes in low- and middle-income countries found nine studies from Africa that met the inclusion criteria, of which three were from West Africa (Ghana, Nigeria and Liberia). Mobile health is defined as “*a medical and public health practice supported by mobile devices such as mobile phones, tablets and other wireless devices*” [49]. The studies showed that despite the potential of mobile health interventions there were gaps in the knowledge base as to how they affect maternal and neonatal health outcomes [50].

### Human resources

Human resources affected the quality of care, accessibility, availability, affordability, acceptability and appropriateness of MNCH services. Conducive factors included successful implementation of strategies and interventions such as task shifting, ensuring organisational environments, climate and cultures that encourage and support performance, and availability of qualified staff and local training institutions. Limiting factors included inadequate staff numbers, inequitable distribution, migration and inadequate resources for training as well as logistics and tools to work with. Closely related to problems with inadequate staff numbers were problems with competence and skills of available staff and the appropriateness or otherwise of capacity-building interventions. This manifested in diverse ways such as in knowledge gaps on obstetric danger signs and when and how to refer clients to the next level [39].

“*There is a proliferation of training institutes but the products from these institutions are no longer graduating with the skills required to make them deliver adequately on the field.*” (Regional level director, Burkina Faso)

Motivation, namely the degree of willingness of health workers [50] to maintain efforts and continous quality improvement [51] towards achieving organisational goals [52–54], was an important conducive or limiting factor. Factors negatively affecting motivation included poor conditions of service, perceived inequity in distribution of incentives, lack of workplace protection, lack of respect and respectful treatment, poor remuneration, non-availability of essential equipment, tools and supplies, and poor work environments. One study reported burnout, expressed as emotional exhaustion and depersonalisation among staff. The same study noted that, despite the challenges, staff still retained a strong sense of accomplishment and confidence in their work [55].

Poor remuneration apart from affecting motivation also led to behaviours that were counter progressive.

“*Due to poor remuneration, most Doctors holding administrative position have become what I call ‘des reunionites’* [regular meeting attenders] *just to get enough perdiem to supplement their meagre salaries, thus delegating their responsibilities to anybody including the less competent personnel.*” (Head of an academic department, Burkina Faso)

On the positive side, and therefore conducive, an opportunity to gain additional education was reported as the most important factor motivating midwifery students in deciding where they would eventually work.

Contextual factors, such as insecurity, conflict and insurgency, affected staff willingness to accept postings and therefore availability, distribution and retention. Migration was fuelled by contextual and health system factors that pushed staff out as well as external factors that pulled staff out.

“*Migration of experts to greener pastures or to where security is more guaranteed or future is better secured.*” (National health director, Nigeria)

### Financing

“*Funds flow is not really regular so at the district and sub-district level they are a bit constrained in implementation and mostly dependent on donor funding. So when there is no donor funds available then service provision is at a standstill.*” (Bilateral development partner, Ghana)

The inadequacy of financing resources to develop and maintain the health system and support service delivery was a problem across all the sub-region. User fees in the form of out-of-pocket payments at point of service use were a common mechanism to try and mobilise the needed money. However, these fees were a limiting factor documented by several studies. They acted as a deterrent to service use, and exposed women and their families to catastrophic expenditures. Several interventions, such as targeted user fee exemptions, community-based health insurance and national health insurance, had been put in place or were being piloted in the sub-region to completely remove or significantly reduce the exposure of mothers and children to these fees.

Several of these interventions had documented positive effects, with evidence of reductions in inequities in access once out-of-pocket user fees were removed. For example, El-Khoury et al. [56] observed that a free delivery and caesarean section policy in Mali had resulted in increased institutional deliveries and needed caesarean sections. In addition, post-caesarean maternal and neonatal deaths declined in most regions from 2006 to 2009, most likely as a result of shorter delays in seeking emergency care and shorter wait times experienced at facilities.

A study in three West African countries (Mali, Senegal and Ghana) found that membership in a community-based health insurance scheme was positively associated with the use of maternal health services. This was particularly so in areas where utilisation rates were very low and for more expensive delivery-related care [57].

Unfortunately, the effect of interventions to remove out-of-pocket fees was often modified by other health system factors such as ability to finance the policy, service availability, perceived quality of service and human resource constraints. The exemption policy for children less than 5 years old in Ghana did not work as designed in part because of failure to reimburse providers in a timely and complete manner [58]. In reaction to reimbursement delays and failures, frontline providers stopped giving exemptions and reinstituted user fees. The NHIS was facing similar problems.

“*People may have the NHIS: I’m sure recently you’ve heard of some facilities that have pulled out of the NHIS because they were not reimbursed, and so they may be registered with the National Health Insurance Scheme but since these facilities will not be offering services, they will not be able to use their insurance for health care. … The scheme actually owes most facilities; they are in arrears. That means that most of these facilities may not be able to purchase most of the things that you probably need for MNCH, so limits their capacity and their ability to deliver very good service to these clients.*” (Health practitioner, Ghana)

The free caesarean section policy in Mali referred to earlier was observed to have inequities in access and utilisation related to service availability with wealthier women making up a disproportionate share of those having free caesareans [56, 59–61]. Women in the richest two quintiles accounted for 58% of all caesareans, while women in the poorest two quintiles accounted for 27%. In rural Mali, some households continued to incur catastrophic health expenditures in accessing maternal health services despite the policy [62]. Living in remote rural areas was associated with the risk of catastrophic spending. Women who underwent caesarean sections continued to incur catastrophic expenses, especially where prescribed drugs were not included in the government-provided caesarean kits.

Fournier et al. [59] observed that, for women living in cities with district hospitals that provided caesarean sections, rates increased from 1.7% before the policy was enforced to 5.7% after 83 months. No significant change in trends was observed among women living in villages with a health centre or no health facility. Abolishing fees for emergency obstetric and newborn care reduced maternal deaths through increased caesarean sections. However, this was not equitable and accessible for those in the rural areas [60].

A KI commented on similar problems with the programme in Burkina Faso.

“*Despite the subsidy system in Burkina Faso, some people are still not able to access caesarean sections in certain regions due their inability to pay for the remaining 20%.*” (Representative of a national professional association, Burkina Faso)

### Information systems

Information and documentation gaps in medical files and records affected the quality of data for decision-making and priority setting. Among the barriers identified to sustaining near-miss audits in Benin were poor or unavailable documentation [63, 64]. The main barriers to the implementation of maternal death reviews in Senegal included poor quality of information in medical files [44].

Timely dissemination and access to and use of information from research and other sources to inform decision-making and implementation were reported as limiting factors.

“*Emerging MNCH related services don’t get introduced until later because national protocols and policy frameworks are not regularly updated to reflect current research finding and WHO standards.*” (Bilateral development partner, Senegal)

Positively, a significant potential to improve access to and use of data for decision-making was reported, and deployment of credible evidence to show policymakers that a problem existed could influence decision-making. A study on agenda-setting in Ghana showed that, where data was available, decision-makers drew on the data to help them frame maternal health problems in a way that got them into and kept them on the agenda [65].

Vital registration systems, despite their importance for monitoring, evaluation and decision-making for MNCH, were under-resourced and poorly implemented.

### Health systems mortar (software)

“*Trust based relationships enhancing community involvement in decision-making is conducive to improving MNCH outcomes. A district medical officer and his collaborators put in place an innovative system for motivating community health workers and have seen a drastic improvement in their MNCH indicators in 2 years. This trust based relationship existing at the community yielded impressive results so we are trying to scale it up to other local health areas.*” (National level director, Ministry of Health, Benin)

Despite the importance of actors, process and power in the functioning of health systems, most of the papers we found were focused on health systems building blocks and/or interventions. The critical role of the mortar often only emerged as a sub-theme. For example, a paper whose primary focus was the evaluation of a task-shifting intervention in Senegal to resolve a long-term shortage of obstetricians (by training district teams consisting of an anaesthetist, general practitioner and surgical assistant in emergency obstetric surgery) encountered several limiting factors related to ‘mortar’. Of the 11 surgical teams trained between 2001 and 2006, only six were functioning in 2006, and the rate of training was not rapid enough to cover all districts by 2015. Reasons included varying and conflicting stakeholder perspectives on the programme, relationships, trust, power and motivation. Central decision-makers considered the policy more viable than training gynaecologists for district hospitals. Senior academic clinicians, on the other hand, resisted the programme. A perceived lack of career progression among the doctors trained and lack of programme coordination were seen as obstacles by these groups. Practitioners felt the work was valuable, but complained of low additional pay and not being replaced during training. Communities appreciated that the services saved lives and money, but called for improved information and greater continuity of care [66].

Information and information asymmetries can be major sources of power or lack of power. Several papers observed that client access to information and use of information acted as part of the conducive or enabling software of health systems. For example, Mills et al. [33] observed, from a study in Northern Ghana, that awareness among women of the policy on free delivery of care at the health facility and of antenatal care services was conducive to utilisation.

Key global actors in health systems development and MNCH in West Africa mentioned by KIs were development partners such as WHO, USAID and UNFPA. These actors and their agents were active at global as well as country level. At the West African sub-regional level, the key actors were seen to be WAHO and the committee or council of ministers. The role of WAHO was described as both political and technical. KIs also mentioned that politicians, especially higher level office holders such as presidents and ministers of health, interface with the sub-regional level in the context of ECOWAS through their interactions with colleague politicians from other countries in the sub-region.

Within countries, the political leadership was described as wielding a lot of power, including the control of resources for health systems and MNCH.

“*… the political leadership, … they wield a lot of power and they control a lot of money and they have a lot of influence when it comes to policy direction.*” (Obstetrician gynaecologist, Ghana)

At the country level, apart from the political leadership, national civil and public service bureaucracy heads such as Chief Directors, Director and Deputy directors were seen as the main holders of power. Non-state actors, such as the Christian Health Association of Ghana, were also mentioned as important at national level. At the sub-national level, frontline provider staff such as doctors, nurses and administrators could wield considerable power often derived from their technical knowledge and control of services and expertise.

“*… health care givers, be it doctors, be it nurses, you know, all the people, pharmacists etc. who work in a health care setting, are also very important when it comes to MNCH.*” (Obstetrician Gynaecologist, Ghana)

Local government, communities and clients were also mentioned by several respondents as key actors with power to affect MNCH outcomes at the sub-national level.

### Context

Multi-level contextual factors emerged from the study as important in health systems and MNCH; these can be clustered as sociocultural, economic, political, ideological, historical, international or global, and challenges with other systems, such as roads and transport, that can directly impact health. These contextual factors acted as conducive and limiting factors to MNCH by their effects on health systems, and on the social determinants of health.

Context inter-related with the health system and its components and caused some of the variability in outcomes. Thus, for example, there were variations in mortality not only by geographic area, but also by socioeconomic and contextual variations such as rural-urban, income quintile, and mother’s education and ethnicity. In one study, socioeconomic status, and religion (Muslim) highly influenced the use of skilled birth attendants [33]. The effects of quality of the road on referral and especially poor road security at night has already been mentioned [48]. Other systems, such as water and sanitation and food and nutrition, also affected MNCH outcomes.

Sociocultural factors included the status of women, and influence of other household and community members on care seeking and decision-making in contexts in which decisions can be the responsibility of members of the extended family rather than the individual. Other factors included low status of women, translated into a lack of control over decision-making, and a low value on girls’ education, which further reinforced domestic power imbalances, and the low status and dependence of women. Literacy (mothers’ education) predicted use of safe motherhood care. Some papers noted that, sometimes, women were expected to justify less obvious needs in an unequal bargaining process with ambivalent recourse opportunities, and might suffer delays in or exclusion from healthcare, low self-esteem and domestic power imbalances. All these translated into effects on service access and utilisation.

Macroeconomic factors affected household access to income as well as the ability to access needed healthcare through their effects on national and household incomes. Political transitions, ideologies, priorities and the availability of champions also affected how MNCH fared.

“*In Ghana there is a lot of political will and commitment* [to the NHIS]*…. There has been a lot of political will, there have been domestic resources that have been mobilized.*” (Bilateral development partner, Ghana)

## Conclusions

Any effort to accomplished the unfinished Millennium Development Goals 4 and 5 agenda in West Africa must recognise the futility of investing mainly in interventions with a relative neglect of the health system and context. It is critical to more effectively span these traditional boundaries and ensure that investments in the three go together with a focus on strengthening health systems and context to enable efficient and effective implementation of proven life-saving interventions. Critical in this will be conceptual models that disrupt the current compartmentalised approach to thinking through the issues and the solutions at global, national and sub-national levels.

**Fig. 2:**
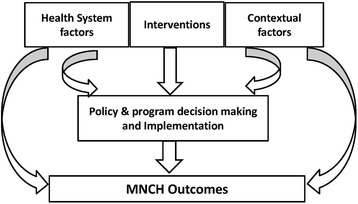
Framework for development and implementation of policies and programmes for improved maternal, newborn and child health outcomes

Drawing on the findings of this study, we propose Fig. 2 as a simple heuristic to guide researchers, decision-makers and implementers in analysis, evidence generation and decision-making to develop more integrated policies and programmes for accelerated improvements in MNCH in West Africa.

Like any other heuristic, Fig. 2 is a practical support to analysis and decision-making rather than a perfect explanatory model. It is a framework whose use will encourage decision-makers at all levels of health systems in sub-Saharan Africa to move away from the current predominant focus on MNCH interventions. Such approaches neglect the conducive and limiting health system and contextual factors that are tightly bound to how policies and accompanying programmes or interventions work in practice, and therefore ultimately their effectiveness, or otherwise, in improving MNCH outcomes.

‘Proven’ or ‘presumed’ effective MNCH interventions are put in place on the assumption that they will improve MNCH outcomes. However, any effects are mediated by mechanisms that are influenced by the decision as to how the intervention is implemented in practice as well as the conditions of the health system in which it is implemented and the context within which the health system operates. Any or all of these factors can act independently as well as in synergy to be conducive or limiting to the implementation processes.

**Fig. 3:**
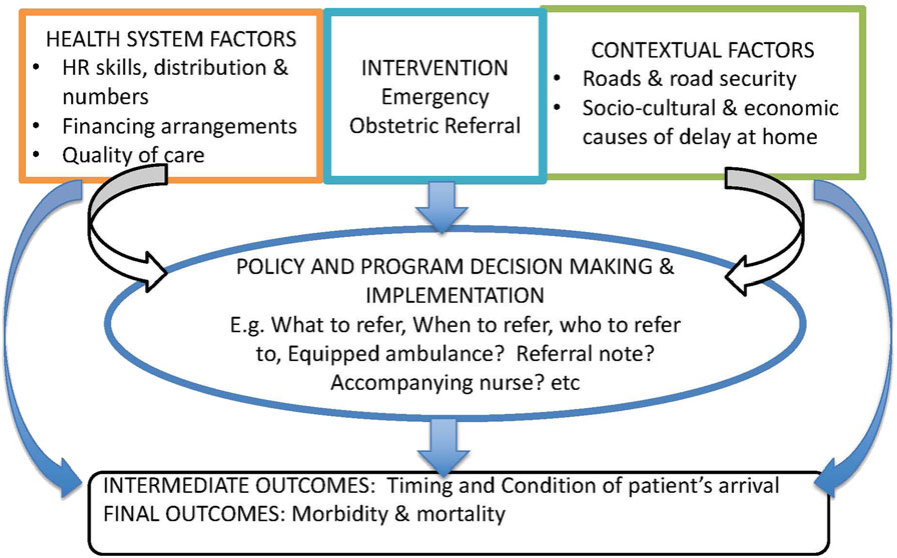
Simple illustration with emergency obstetric referral

A simple illustration of analysis drawing upon this framework is show in Fig. 3 using emergency obstetric referral.

**Fig. 4:**
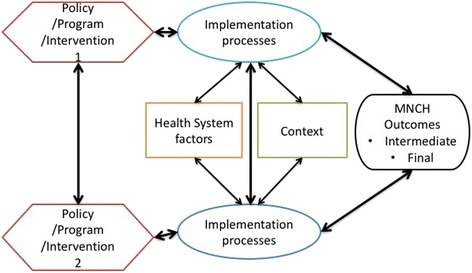
Superimposing complexity: multiple interacting programmes

In health systems, several interventions are simultaneously in place. Thus, there are several cycles interacting with and influencing each other, further compounding the complexity. This is illustrated, still as a simplification, in Fig. 4.

### Limitations of the study

We had to exclude the Portuguese language literature for practical reasons related to the cost of inclusion. This raises the possibility that we may be missing differences between the Lusophone countries of Cape Verde and Guinea Bissau and the rest of the ECOWAS in our analysis. Secondly, our study was entirely qualitative and we are unable to provide any empirical data on the magnitude of our observations across West Africa. This remains part of the agenda for future research. However, it is worth noting that not all the conducive and limiting factors raised in this paper lend themselves to quantification; values, power and process in health systems and their role as conducive and limiting factors to MNCH improvement are not necessarily quantifiable. The research agenda for these factors will be the need for more qualitative research to improve understanding.

## Abbreviations

ECOWAS: Economic Community of West African States
KI: key informant
MNCH: maternal, newborn and child health
NGO: Non-governmental organisation
NHIS: National Health Insurance Scheme
PHC: primary healthcare
WAHO: West African Health Organization
